# Cytomegalovirus Hepatitis During Pregnancy

**DOI:** 10.1155/S1064744995000524

**Published:** 1995

**Authors:** Ying Chan, Maria Kean-Chong, David Gonzalez, Joseph Apuzzio

**Affiliations:** Department of Obstetrics and Gynecology University of Medicine and Dentistry of New Jersey New Jersey Medical School 185 South Orange Avenue Newark NJ 07103 USA

## Abstract

*Background:* Although cytomegalovirus (CMV) is an uncommon cause of viral hepatitis during pregnancy, a definitive diagnosis is important because of the potential for congenital CMV. In the case reported here, a diagnosis of hepatitis caused by CMV was made after the more common viral pathogens had been ruled out.

*Case:* A 17-year-old, 12-week pregnant patient was evaluated for fever and right upper quadrant tenderness. A serologic evaluation revealed elevated liver function levels and a positive maternal serology for CMV IgM. A diagnosis of hepatitis caused by CMV was made after the more common viral pathogens and drug-induced hepatitis had been ruled out. She was counseled about the potential effects of CMV on her fetus.

*Conclusion:* A step-wise approach to the diagnosis of viral hepatitis during pregnancy is needed to determine the etiology because a potential teratogenic virus may be involved.


Infectious Diseases in Obstetrics and Gynecology 3:164–165 (1995)
© 1995 Wiley-Liss, Inc.

### Cytomegalovirus Hepatitis During Pregnancy

**Ying Chan, Maria Kean-Chong, David Gonzalez, and Joseph Apuzzio**

*Department of Obstetrics and Gynecology, University of Medicine and Dentistry of New Jersey, New Jersey Medical School, Newark, NJ*

ABSTRACT

***Background:*** **Although cytomegalovirus (CMV) is an uncommon cause of viral hepatitis during pregnancy, a definitive diagnosis is important because of the potential for congenital CMV. In the case reported here, a diagnosis of hepatitis caused by CMV was made after the more common viral pathogens had been ruled out.**

***Case:*** **A 17-year-old, 12-week pregnant patient was evaluated for fever and right upper quadrant tenderness. A serologic evaluation revealed elevated liver function levels and a positive maternal serology for CMV IgM. A diagnosis of hepatitis caused by CMV was made after the more common viral pathogens and drug-induced hepatitis had been ruled out. She was counseled about the potential effects of CMV on her fetus.**

***Conclusion:*** **A step-wise approach to the diagnosis of viral hepatitis during pregnancy is needed to determine the etiology because a potential teratogenic virus may be involved.** © 1995 Wiley-Liss, Inc.

KEY WORDS

Infectious diseases, teratology, prenatal diagnosis

Viral hepatitis is one of the more common medical complications of pregnancy. The most common viruses causing viral hepatitis in the United States are hepatitis B, which is responsible for about 80% of all cases of hospitalized pregnant women, and hepatitis A, which is responsible for about 10% of cases. Less common viral agents causing hepatitis include hepatitis C, cytomegalovirus (CMV), Epstein-Barr virus, coxsackie B virus, and herpes. The first approach to diagnosing a patient with suspected hepatitis is to obtain a detailed history to elicit risk factors. These risk factors include a recent blood transfusion, person in the household with hepatitis, consort with hepatitis, and environmental exposure as in health-care workers or day-care workers. A step-wise approach to ordering laboratory tests for viral hepatitis is imperative, since a diffuse approach may be expensive and unnecessary. Reported here is a case of viral hepatitis caused by CMV during pregnancy.

### CASE REPORT

A 17-year-old $G_1P_0$, approximately 12 weeks pregnant, presented to the emergency room with complaints of back pain, fever, right upper quadrant pain, and malaise for the 2 previous days. She denied sore throat, neck pain, cough, shortness of breath, chest pain, and diarrhea. Her medical history was negative for diabetes, hypertension, and heart, liver, lung, or gastrointestinal disease. The patient denied having eaten raw shell fish or having had contact with a person in the household or a consort with hepatitis. She denied substance abuse and reported that she did not take any medications.

The patient denied any surgical history or known allergies. The family history was negative. The

**Address correspondence/reprint requests to Dr. Joseph Apuzzio, Department of Obstetrics and Gynecology, University of Medicine and Dentistry of New Jersey, New Jersey Medical School, 185 South Orange Avenue, Newark, NJ 07103.**

Received April 11, 1995

Accepted August 1, 1995

physical examination revealed a well-developed, well-nourished female. She did have tenderness in the right upper quadrant with voluntary guarding, but she had no rebound tenderness. The uterus was 12 weeks in size. The serologic markers for hepatitis A and hepatitis B surface antigen and antibody were negative. A mononucleosis spot test was negative. The liver function studies were mildly elevated, with the SGOT 154 (normal up to 40), the SGPT 125 (normal up to 35), and total bilirubin 0.7. The CMV titers were positive for IgG and IgM, indicating a recent infection. While she was in the hospital, the SGOT increased to 302 and the SGPT to 135, and the GGTP was 170 (normal, 5–55). The hepatitis C antibody was negative. In view of her history as well as the laboratory findings, we felt that the diagnosis was CMV hepatitis. In addition, since this infection appeared to be of recent onset and carried with it the risks of CMV for the unborn fetus, we recommended obtaining a prenatal diagnosis with amniocentesis and viral culture of the amniotic fluid. We also recommended follow-up ultrasound scans for fetal growth and structural malformations. The patient declined amniocentesis. Since the pregnancy was unplanned, she elected to terminate it.

### DISCUSSION

Viral hepatitis is a common medical complication during pregnancy, but hepatitis caused by CMV is uncommon, possibly because many patients who have hepatitis either have mild symptomatology and do not seek medical attention or they do not receive the appropriate serologic studies for CMV. The above case demonstrates that, when the more common causes of hepatitis such as a drug reaction according to the medical history or hepatitis B and hepatitis A according to serologic tests have been ruled out, the less common causes of viral hepatitis should be determined serologically. Determining the cause is particularly important during pregnancy since other viral causes of hepatitis such as CMV can cross the placenta to infect the fetus. In fact, CMV is the most common cause of congenital infection, affecting 30,000–40,000 liveborn infants annually in the United States.[1–3] It is estimated that CMV will be transmitted to 50% of fetuses after the primary infection and that 10% of these infected infants will be clinically affected. An excellent review by Duff[4] covers the effects of CMV on the fetus.

A step-wise progression of ordering tests for the more common viral agents responsible for hepatitis will lead to the diagnosis. The medical history is an important guide to testing since the risk factors for hepatitis will help the health-care provider determine which test should be ordered first. For example, a patient who has recently consumed raw shell fish is likely to have hepatitis A; therefore, a serology for hepatitis A virus IgM should be ordered. Because our case had no identifiable risk factor, we initially ordered serologic studies for hepatitis A and hepatitis B. Since these tests were negative, we ordered tests for hepatitis C antibody and CMV IgG and IgM. This progression of tests allowed us to use the laboratory resources in a more cost-effective manner.

When possible, one should seek a definitive diagnosis of CMV infection by means of a viral culture. For example, in our case, the amniotic fluid or abortus material could have been cultured. Unfortunately, the patient and her private physician did not allow us to perform these tests. Positive cultures for CMV from this material would have documented a maternal CMV infection. However, negative cultures would not have ruled out a maternal infection since CMV infection is transmitted to the fetus in only 40–50% of cases.

### REFERENCES

1. Freij BJ, Sever JL: Herpesvirus infections in pregnancy: Risk to embryo, fetus, and neonate. Clin Perinatol 15: 203–231, 1988.
2. Alford CA, Britt WJ: Cytomegalovirus. In: Fields BN, Knipe DM (eds): Virology. 2nd ed. New York: Raven Press, pp 1981–2010, 1990.
3. Makkon M, Huttumen M, Martikaimen A, Saarikoski S: Cytomegalovirus hepatitis late in pregnancy. Int J Gynecol Obstet 37:199–201, 1992.
4. Duff P: Cytomegalovirus infection in pregnancy. Infect Dis Obstet Gynecol 2:146–152, 1994.
